# CT imaging of peritoneal carcinomatosis with surgical correlation: a pictorial review

**DOI:** 10.1186/s13244-021-01110-6

**Published:** 2021-11-12

**Authors:** Panagiota Berta Panagiotopoulou, Nikos Courcoutsakis, Apostolos Tentes, Panos Prassopoulos

**Affiliations:** 1 Department of Radiology, University Hospital of Alexandroupolis, Democritus University of Thrace, Alexandroupolis, Greece; 2 Department of Surgery, Euromedica “Kyanos Stavros” Hospital, Thessaloniki, Greece; 3 Department of Radiology, AHEPA UniversityHospital, Aristotle University of Thessaloniki, Thessaloniki, Greece

**Keywords:** Peritoneal carcinomatosis, Cytoreductive surgery, Computed tomography

## Abstract

Cytoreductive surgery in combination with hyperthermic intraperitoneal chemotherapy has revolutionized the survival and the quality of life in selected patients with peritoneal carcinomatosis. Preoperative CT is important for the selection of patients that may benefit from cytoreductive surgery and is useful for surgical planning. There are several tasks for the radiologist during CT interpretation: to describe cancerous implants on a “site-by-site” basis in the peritoneum, ligaments, mesenteries and visceral surfaces, to analyze patterns of involvement and to estimate the disease burden. Knowledge of the correlation between the CT and the surgical findings enhances the understanding of the disease and facilitates the communication between radiologists and surgeons.

## Key points

- CT imaging findings correlate with findings at surgery in patients with PC.
- CT findings in PC should be reported on a “site-by-site” basis.
- CT findings in PC help to choose candidates for cytoreductive surgery.

## Introduction

The peritoneal cavity is a common site of metastatic spread from intraabdominal malignancies, mainly ovaries, large bowel, stomach and pancreas [1, 2]. Traditionally, peritoneal carcinomatosis has been regarded as an end-stage disease with very poor prognosis, as most patients died within 6 months after diagnosis and only palliative treatment was applied [3–5]. New aggressive therapeutic approach, namely cytoreductive surgery (CRS) in combination with hyperthermic intraperitoneal chemotherapy (HIPEC) is an evolving treatment option and is associated with an improved survival rate in selected patients [4, 6, 7]. The aim of the procedure is to achieve complete excision of all visible neoplastic tissue from the peritoneal cavity; this may require both peritonectomy procedures and visceral resections, in a number of patients. The CRS is combined with HIPEC, yielding a high local drug concentration; hyperthermia—enhancing the cytotoxicity of anticancer drugs- facilitates eradication of any residual microscopic disease [4, 8, 9]. High morbidity and cost of this method require accurate patient selection to achieve optimal results [5, 6]. The number, exact localization and distribution of peritoneal implants are prognostic factors for long-term outcomes and they influence the likelihood of complete cytoreduction. For example, extensive involvement of the small bowel or mesenteric root decreases the chance for optimal cytoreduction, as enough small bowel needs to remain in place, to avoid the “short bowel syndrome” [5, 10].

Multidetector computed tomography (MDCT) is the most commonly used technique for the detection of peritoneal carcinomatosis and for the evaluation of the extend of the disease [6, 11, 12], due to availability, low cost and short execution time. The acquisition of thin slices gives the ability to perform high quality multiplanar reconstructions (MPR), allowing for better demonstration of lesions in specific anatomic areas, as the subdiaphragmatic space and the pelvis [10, 13–15].

The aim of this pictorial review is to correlate the radiological with the surgical findings to deepen the understanding of imaging in the era of cytoreductive surgery and to enhance the communication between radiologists and surgeons.

### CT technique

CT imaging is performed with the patient in the supine position from the distal thorax to the inguinal region with the following parameters for a 64-slice CT equipment:

collimation 64X0.625 mm, pitch 0.984:1, rotation time 0.5 s, speed 39.37 mm/rotation, kV 120, smart mA 120–450 and a noise index between 10 and 12. Reconstruction standard soft tissue filter, coronal/sagittal images (section thickness 3 mm, interval 2 mm).

The coverage of the lung bases aids in the assessment of epiphrenic lymph nodes. MDCT scanning is performed after intravenous administration of contrast medium, with flow rate of 3 ml/sec, during the portal venous phase [16]. In routine MDCT a solution of 800–1000 ml iodinated contrast medium (positive contrast) was administered per os 35–45 min before examination for bowel distention and opacification. In CT-Enteroclysis (CTE), a solution of 1800–2000 ml polyethylenglycol was used as enteral contrast through a nasojejunal catheter (negative contrast). Shortly before data acquisition, 20 mg of an antiperistaltic agent (scopolaminbutylbromid, Buscopan, Boheringer Ingelheim, Basel, Switzerland) was injected intravenously to diminish bowel motion and related artifacts in both MDCT and CTE [5, 16].

### CT evaluation

Knowledge of the pathophysiologic mechanism of the tumor spread and the most common sites and forms of peritoneal involvement guides the radiologist to the correct interpretation of the CT examination.

Distribution of PC is influenced by the circulation and resorption of peritoneal fluid. Peritoneal impants usually develop in anatomic locations where ascites pools and in the sites of maximal resorption [10, 17, 18].

As Meyers introduced [19], ascites from the right infracolic space moves along the small bowel mesentery to the pouch at the ileocecal junction, where it is temporarily arrested. In the left infracolic space, the fluid flows to the surface of the sigmoid mesocolon, which stops a quantity of fluid. Consequently, ascitic fluid gravitates to the most dependent area within the pelvis, namely the pouch of Douglas and, then, to the lateral paravesical spaces. Due to the negative pressure under the diaphragm during expiration, fluid moves in a cranial direction via the paracolic gutters. There is preferential flow of the ascitic fluid along the right paracolic gutter, which is wider and communicates freely with the right subhepatic and right subphrenic space. Ascitic fluid flows in a lesser extend along the left paracolic gutter, which is swallow and does not communicate freely with the left subdiaphragmatic space, due to the presence of the phrenicocolic ligament [19].

The resorption of peritoneal fluid mainly occurs through the greater omentum and the subdiaphragmatic space [4, 10].

Accordingly, the most common sites of involvement in peritoneal carcinomatosis are presented in Table 1.

**Table 1:** Common sites for peritoneal implants

Gravity depended areas—areas of arrested flow	
Pouch of Douglas—rectouterine/retrovesical space	
Right lower quadrant—ileocecal region	
Left lower quadrant—superior aspect of sigmoid	
Right paracolic gutter	
Pouch of Morrison	
Areas of fluid resorption	
Greater omentum	
Right subdiaphragmatic space	

MDCT accuracy for the detection of peritoneal implants varies with their location and is higher along the gutters and over the free surface of the liver and spleen, and lower in the small bowel and its mesentery. Detection of peritoneal implants is facilitated by the presence of ascites. Overall diagnostic accuracy of 94%, specificity of 92% and sensitivity between 75 and 81% have been reported for MDCT [2].

Preoperative MDCT evaluation of the distribution and burden of carcinomatosis are of decisive significance in the selection of patients who could benefit from cytoreduction and are important information for surgical planning [20]. Form and size of peritoneal implants should be reported in every region of the peritoneal cavity. A road map for the radiologist might be the surgical evaluation of malignant burden in the peritoneal cavity, employing the peritoneal cancer index (PCI).

**Fig. 1:**
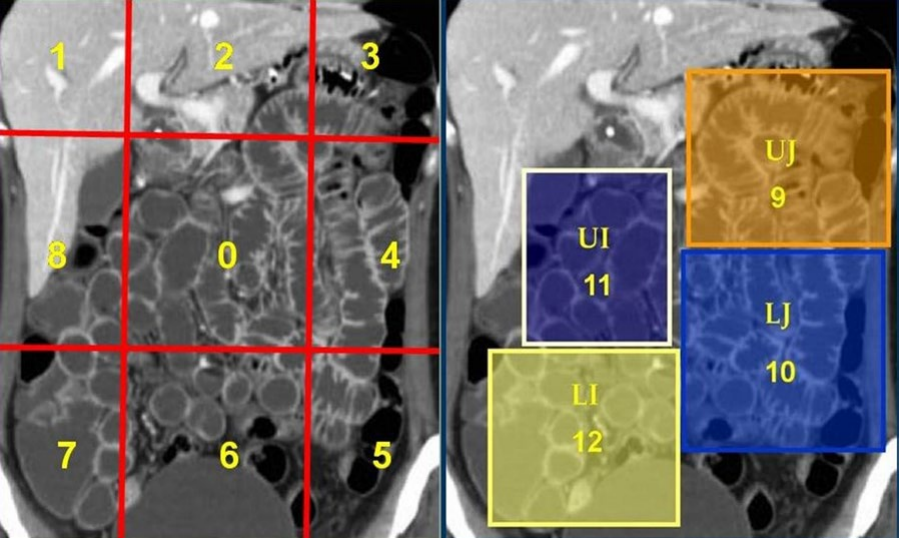
Sugarbaker’s peritoneal cancer index (PCI) divides the peritoneal cavity into 9 abdominopelvic regions (0 central, 1 right hypochondrium, 2 epigastrium, 3 left hypochondrium, 4 left paracolic gutter, 5 left iliac fossa, 6 pelvis, 7 right iliac fossa, 8 right paracolic gutter) and 4 enteric regions (9 upper jejunum, 10 lower jejunum, 11 upper ileum, 12 lower ileum)

PCI, introduced by Jacquet and Sugarbaker [21], is a scoring system for the assessment of both cancer distribution and cancer implant size in 9 abdominopelvic regions and 4 enteric regions (Fig. 1), and it is calculated intraoperatively. In each region the largest diameter of the implants is measured and graded as follows: 0 grade for no visible tumor, 1 grade for larger lesion diameter < 0.5 cm, 2 grades for lesion diameter between 0.5 and 5 cm and 3 grades for lesion diameter > 5 cm or for confluent lesions. The sum of the scores in all 13 regions constitutes the PCI, which may vary between 0 and 39. Similarly, the CT based PCI (CT-PCI) can be calculated preoperatively, by applying the same principals as the surgical PCI [7, 20, 22]. It has been proposed to include in the radiology report (i.e., in the form of a template) the assessment of disease burden in each one of the thirteen abdominal segments in accordance with the division applied by surgeons to calculate the PCI score (Fig. 1) [23]. The so formed radiological PCI correlates well with surgical PCI [24, 25]. The use of radiological PCI score facilitates the communication between radiologists and surgeons and it could be useful for surgical planning [26, 27]. If the CT-PCI is 20 or greater, the likelihood of complete cytoreduction is minimized. Moreover, involvement of jejunal regions 9 and 10 has more unfavorable prognosis than involvement of ileal regions 11 and 12 [20, 22].

MDCT is the most widely applied imaging modality for preoperative evaluation in PC. The Peritoneal Surface Malignancy Group has accepted CT as the fundamental imaging modality in the preoperative selection process [11]. MRI has slightly better sensitivity than MDCT and is considered more accurate in the subdiaphragmatic areas [23, 28]. On the other hand, PET/CT is recommended to assess disease relapse after treatment due to its excellent specificity [23, 27, 28].

Concerning the role of preoperative laparoscopy, conflicting views have appeared in the literature. A recent article concluded that both laparoscopy and CT are equally effective in the preoperative PC categorization [29]. .

Several concerning radiologic features may be associated with an increased incidence of incomplete cytoreduction; they may indicate either unresectability, or require complex resections that are associated with increased morbidity and mortality. Imaging findings associated with incomplete cytoreduction include: extensive small bowel involvement, mesentery drawn together by tumor (“clumped bowel”), mesenteric or para-aortic lymphadenopathy, obstructed ureter, psoas muscle invasion, pelvic side wall invasion, involvement of the hepatoduodenal ligament, presence of large amount of tumor in the gastrohepatic ligament and/or gastric outlet obstruction due to tumor encasing the antrum of the stomach [30, 31].

### Imaging findings and patterns of peritoneal carcinomatosis morphology of the peritoneal implants

**Fig. 2:**
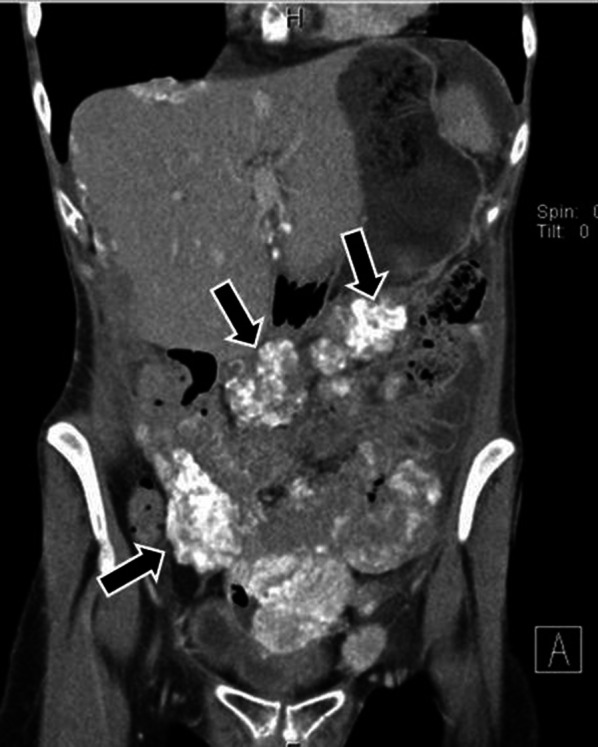
Patient with ovarian cancer and peritoneal carcinomatosis. Coronal reconstructed CT image shows multiple voluminous calcified implants in the peritoneum (black arrows)

Peritoneal implants are solid with heterogeneous enhancement in the vast majority of cases and may have the form of nodules, plaques or masses [7]. Occasionally multiple tiny implants may be manifested as fat stranding. Miliary tiny implants covering the parietal peritoneum may be seen as thickening and enhancement of parietal peritoneum. Peritoneal implants may rarely be cystic, especially from mucinous carcinoma of the ovary or colon, thus mimicking loculated fluid [31]. Calcifications of peritoneal implants are sometimes observed and suggest that the primary site is either serous ovarian cystadenocarcinoma or gastric carcinoma (Fig. 2). Calcification may also occur as a consequence of chemotherapy [10, 23].

### Ascites

Ascites is a common and early sign of PC and when present, it facilitates the detection of peritoneal deposits. Neoplastic ascites may be due to increased capillary permeability and fluid production or to obstructed lymphatic vessels and decreased absorption [32]. Ascites can be either diffuse or loculated/ septated due to adhesions; the latter may present as a cystic lesion with mass effect [31].

### Peritoneum and ligaments

**Fig. 3:**
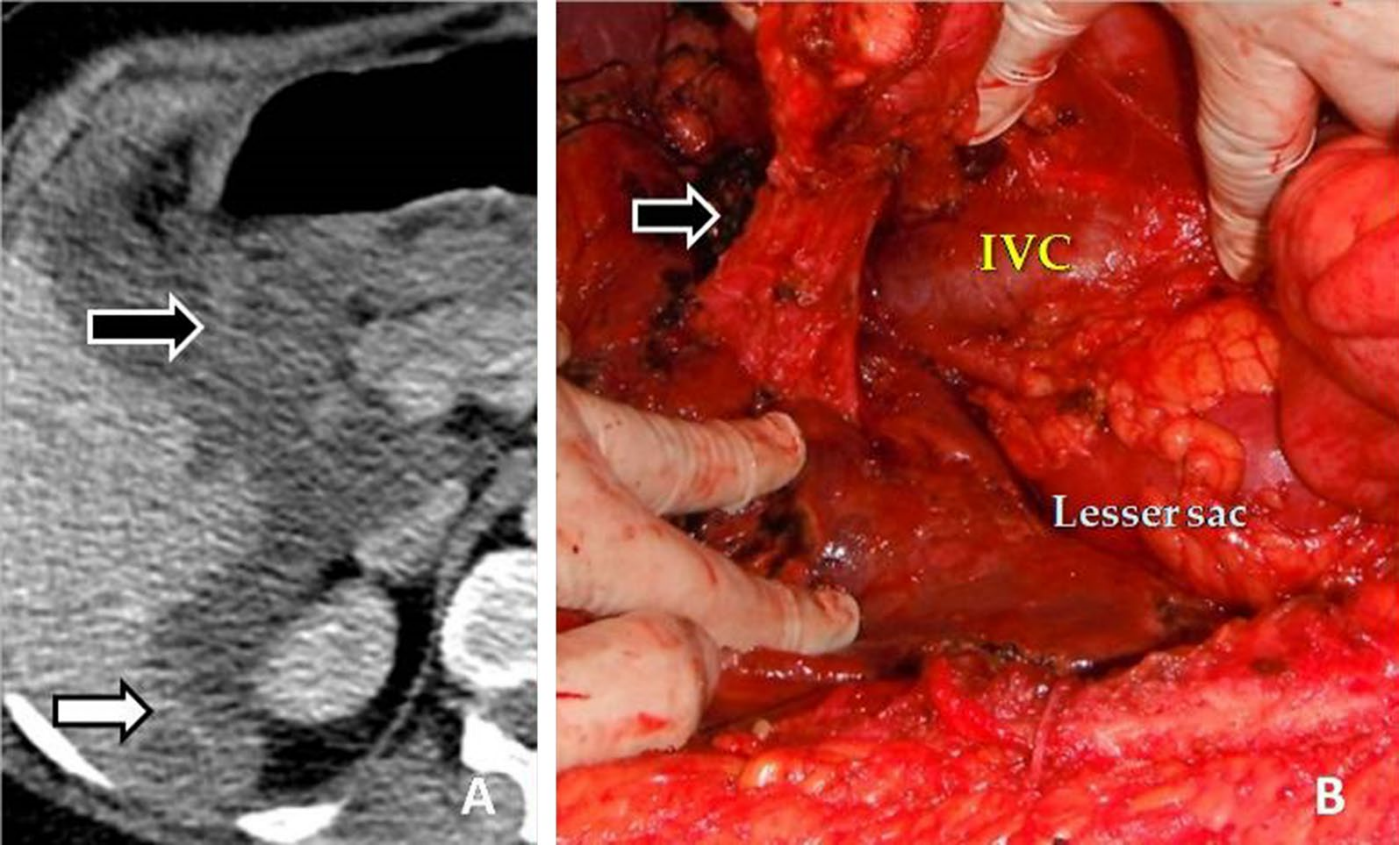
**a** Εnhanced CT image of a patient with peritoneal carcinomatosis demonstrates extensive infiltration of the hepatoduodenal ligament (black arrow) and implants in the Morrison pouch (white arrow). **b** At surgery, malignant implants are demonstrated at the hepatoduodenal ligament (arrow). Inferior vena cava (IVC) and lesser sac are also seen

**Fig. 4:**
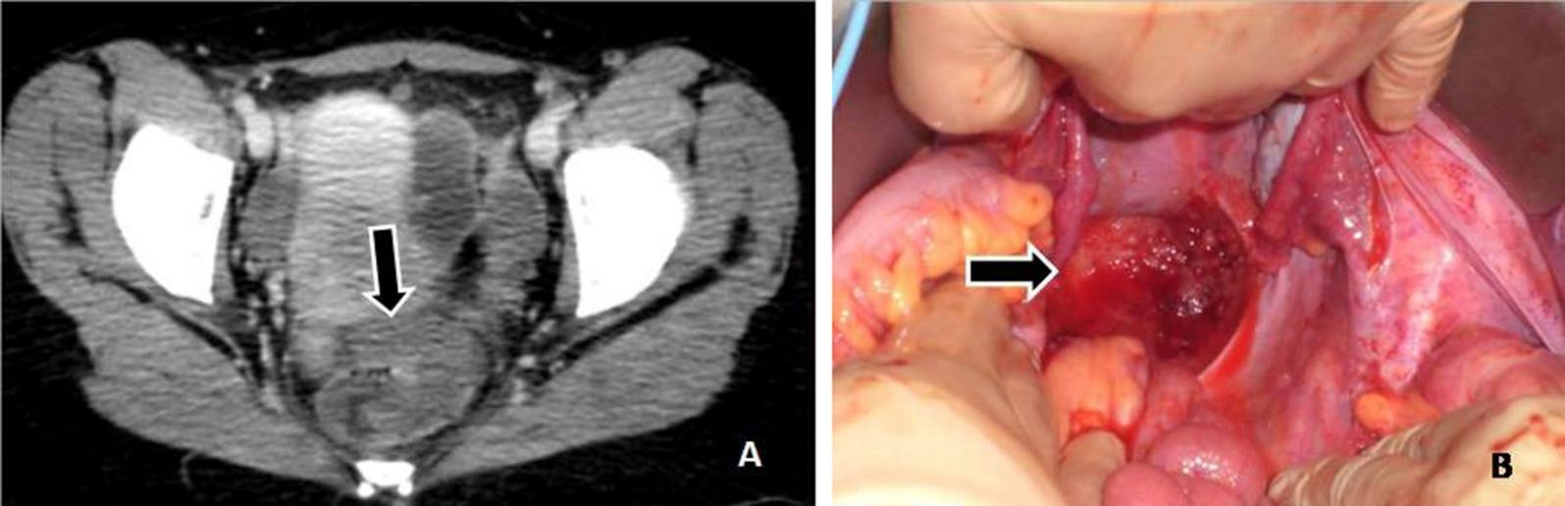
**a** An enhancing mass is demonstrated in the pouch of Douglas (arrow). **b** The lesion as seen at surgery (arrow)

**Fig. 5:**
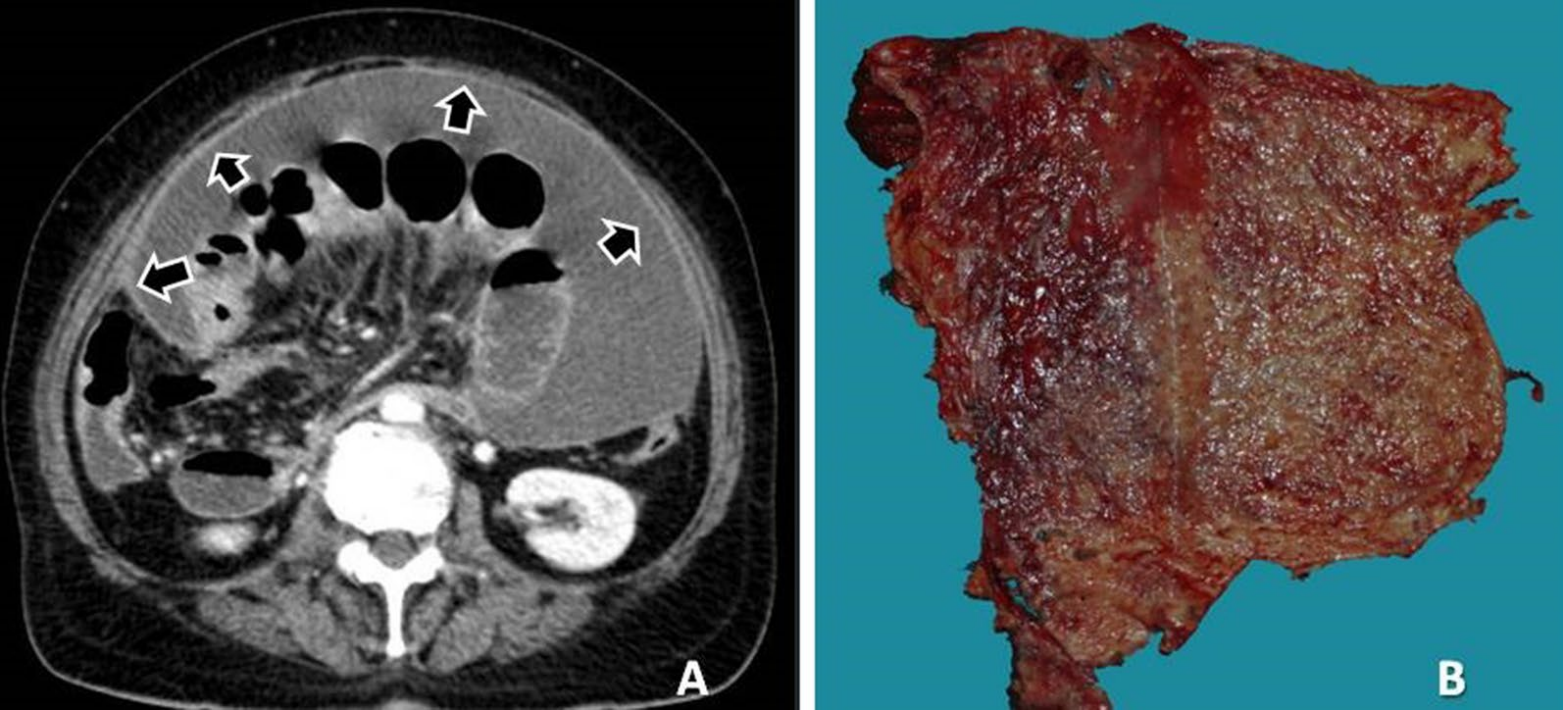
**a** CT enhanced image delineates thickened and enhanced peritoneum (arrows) and ascites. **b** Surgical specimen of the excised peritoneum

**Fig. 6:**
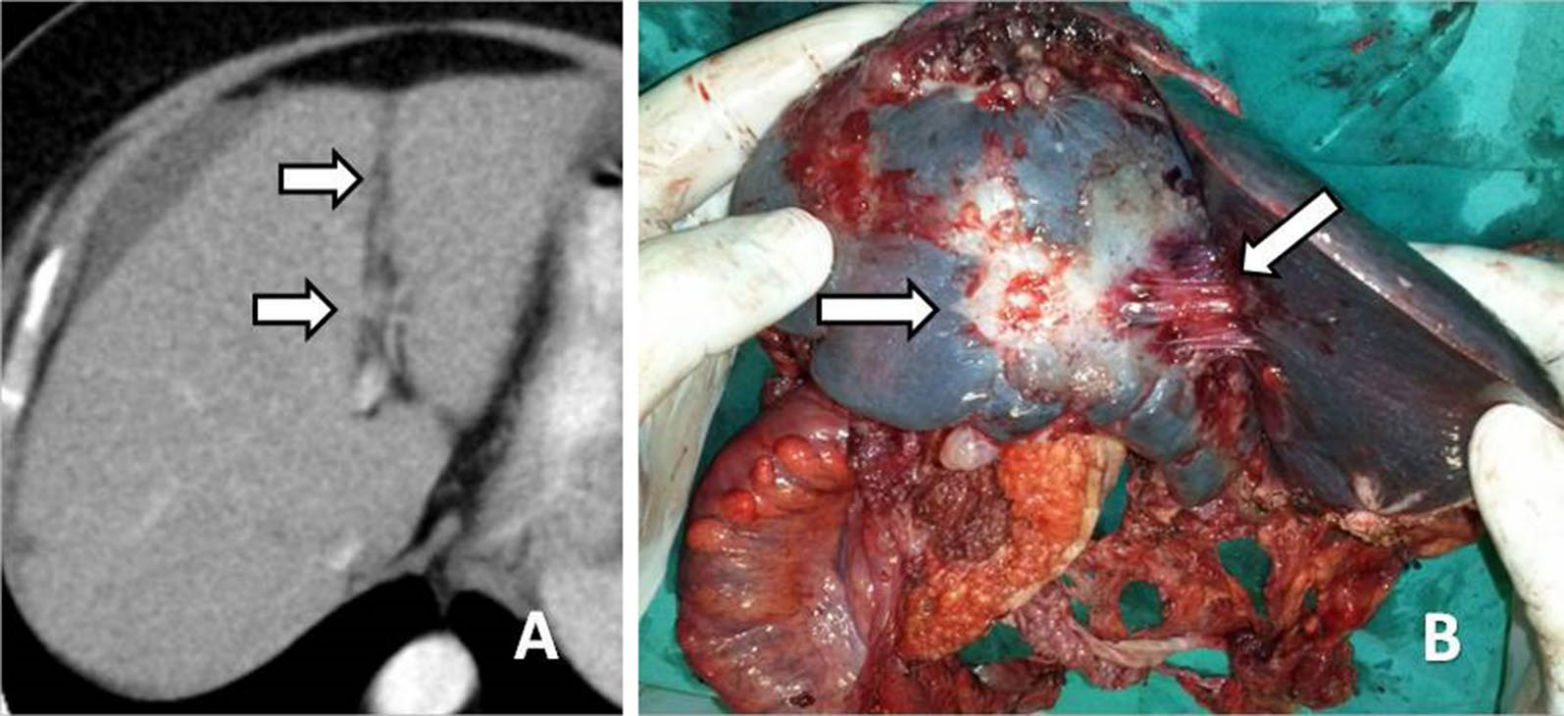
Patient with ovarian cancer and peritoneal carcinomatosis. **a** Abnormal tissue, in a “plaque-like” form, is recognized at the area of the falciform ligament (white arrows). Small perihepatic ascites is also seen. **b** Malignant deposits are demonstrated in the falciform ligament (white arrows)

Metastatic deposits to the peritoneum/ligaments (Fig. 3) may be manifested either as nodules or masses (Fig. 4) or thickening and enhancement of the peritoneum (Fig. 5) or discrete plaques or diffuse plaque-like coating the peritoneum (Fig. 6) [10, 17].

### Surface of intraperitoneal organs

**Fig. 7:**
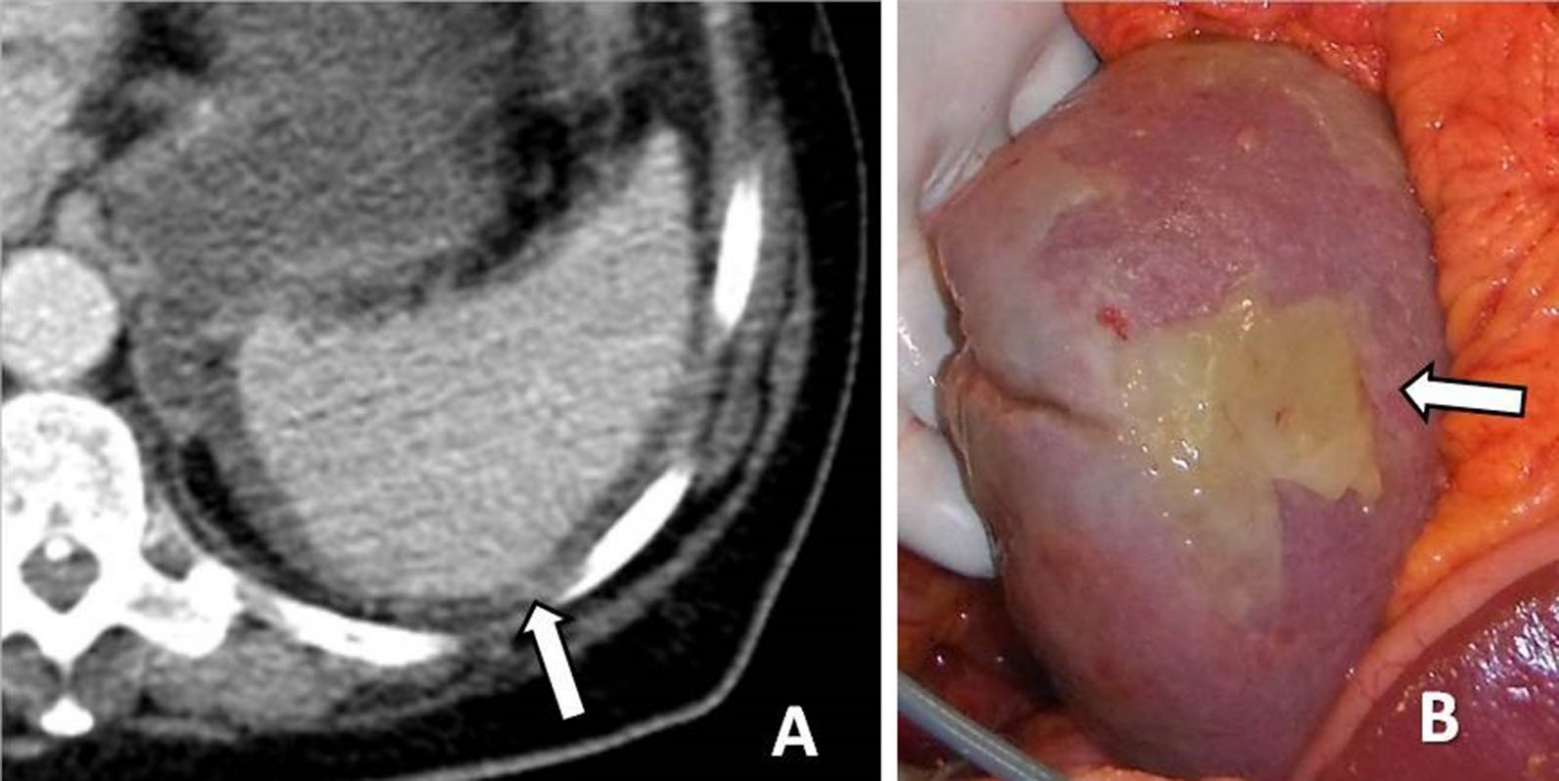
Patient with pseudomyxoma peritonei. **a** Enhanced CT image shows perisplenic myxomatous ascites and deposits on the splenic surface (arrow). **b** The surgical specimen verifies the myxomatous implants on the surface of the spleen (arrow)

Metastatic deposits on the visceral surface of intraperitoneal organs may present as plaques, nodules or masses (Fig. 7). When carcinomatous implants cover the peritoneal surface of the liver or spleen, they may indent the parenchyma creating a “scalloping” appearance [10, 33].

### Greater omentum

**Fig. 8:**
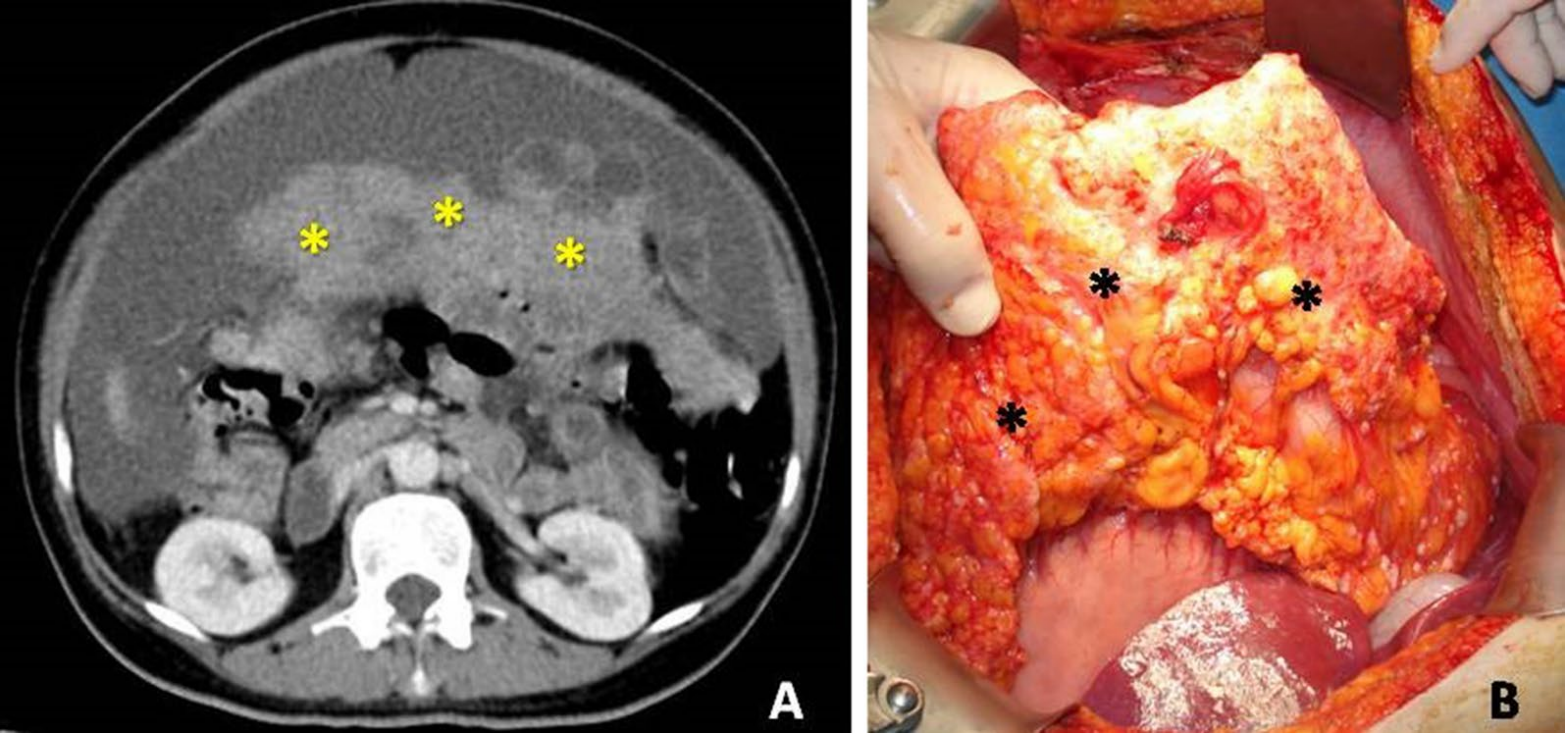
**a** Contrast-enhanced CT image reveals massive infiltration of the greater omentum—“omental cake” (asterisks). **b** At surgery extensive infiltration of the omentum is demonstrated (asterisks)

The greater omentum is a very common site of involvement in peritoneal carcinomatosis, especially from ovarian primary tumors. Early involvement of the omentum is presented as increased fat attenuation, fine reticulonodular pattern or nodules. In advanced stages the deposits range from discrete masses to generalized invasion of the omentum, which takes the form of a thick soft tissue plaque the so called "omental cake" (Fig. 8) [10, 18].

### Small bowel and small bowel mesentery

**Fig. 9:**
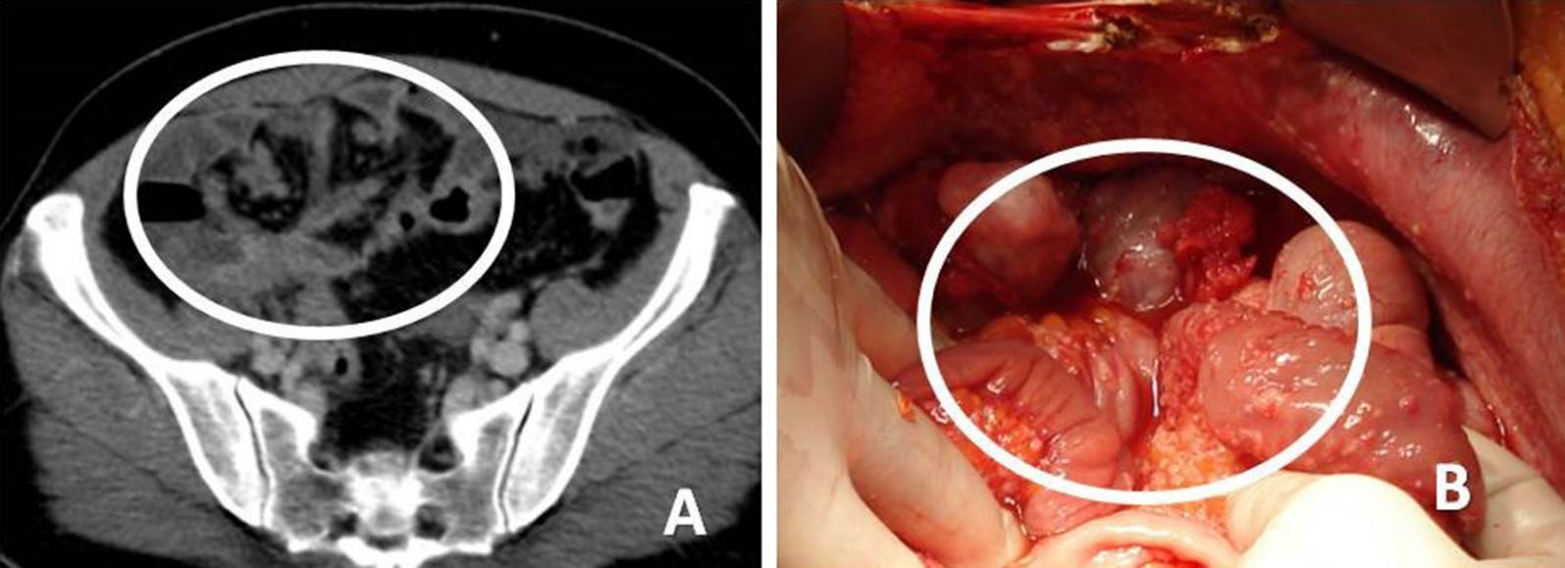
**a** Contrast enhanced CT enteroclysis image shows non dilated intestinal loops due to rigidity caused by multiple tiny implants on the surface of the intestinal loops (white circle). **b** At surgery shorted and distorted intestinal loops with numerous deposits are seen (white circle)

**Fig. 10:**
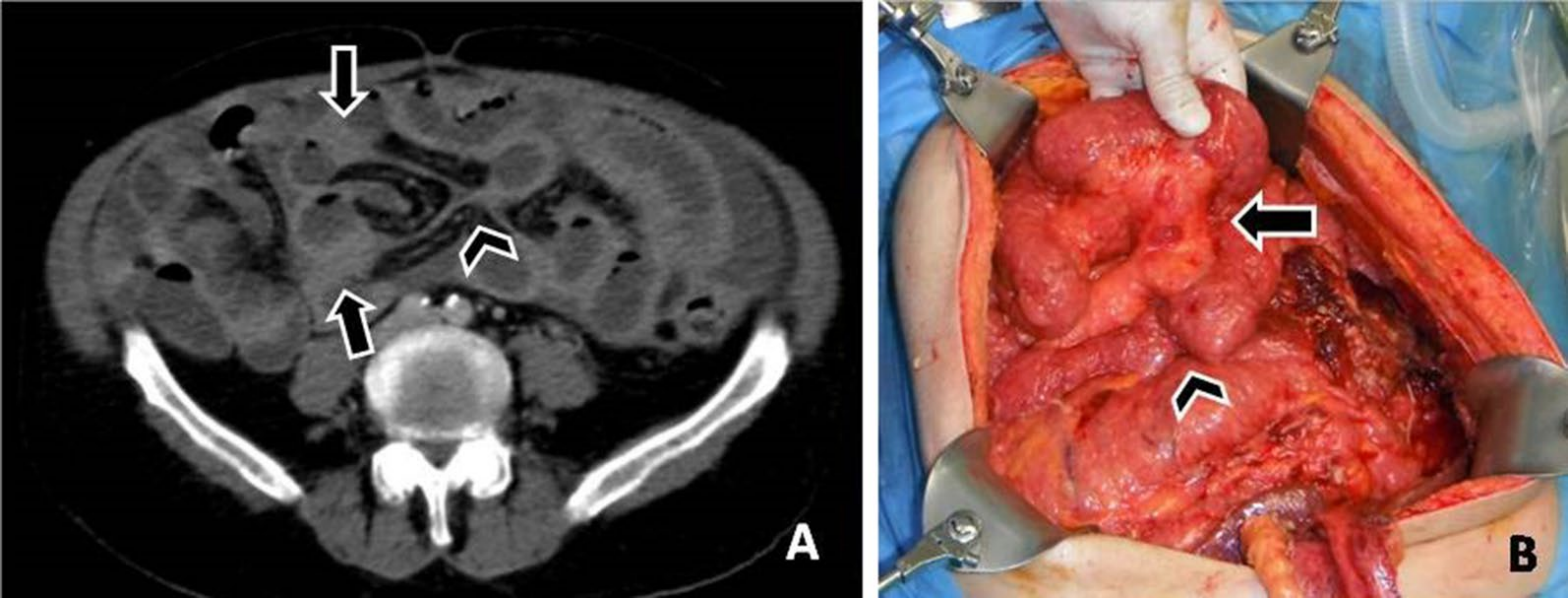
**a** CT enteroclysis image demonstrates extensive wall thickening of the small bowel (arrows)—“layered type” of SB involvement and thickening of the mesenteric surface (arrowhead). **b**. Corresponding surgical imaging

The extend of the disease in the small bowel (SB) and its mesentery plays a pivotal role in the selection of patients for cytoreductive surgery. Enough SB length should be remain in place to allow for adequate oral nutrition. Thus, the evaluation of SB and SB mesentery is crucial in the preoperative imaging assessment. In conventional MDCT, implants attached to collapsed or partially distended intestinal loops may be difficult to be noticed. Adequate SB loop distention is mandatory for the depiction of small implants on the intestinal wall. CT combined with CTE can achieve sufficient small bowel distention by administration of high volume of negative contrast medium [5]. CTE has been proposed in the preoperative work-up in candidates for cytoreduction, since it is able to reveal small separate or coalescent implants on the intestinal wall/mesentery [5, 34]. Implants located on the small bowel wall may manifest on CT as nodules, masses between small bowel loops or masses that adhere to neighboring small bowel loops and may result in bowel obstruction. Multiple tiny implants covering the surface of the SB loops may be seen as wall thickening and enhancement, restricted distensibility and distortion of SB segments (Fig. 9) with wall irregularity or intestinal stenosis. This specific type of SB involvement has been described as “layered-type” form of peritoneal carcinomatosis [5, 10] and corresponds to extensive coating of intestinal loops by thin cancerous plaques (Fig. 10). It is considered a major contraindication for surgery [35, 36].

**Fig. 11:**
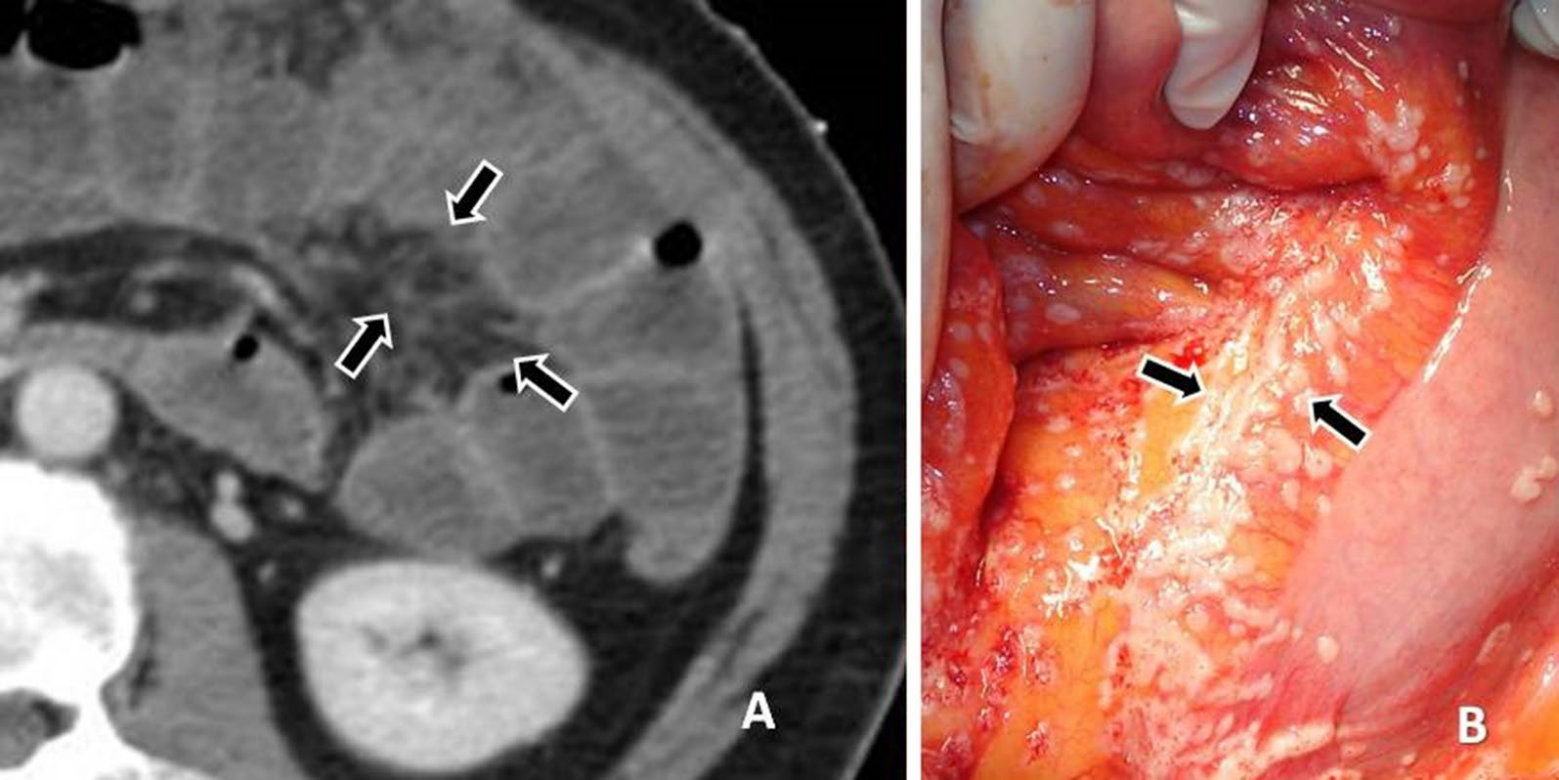
**a** CT enteroclysis image discloses increased attenuation and stranding of the mesenteric fat (arrows). **b** At surgery the CT finding is produced by multiple tiny implants on the small bowel mesentery (arrows)

**Fig. 12:**
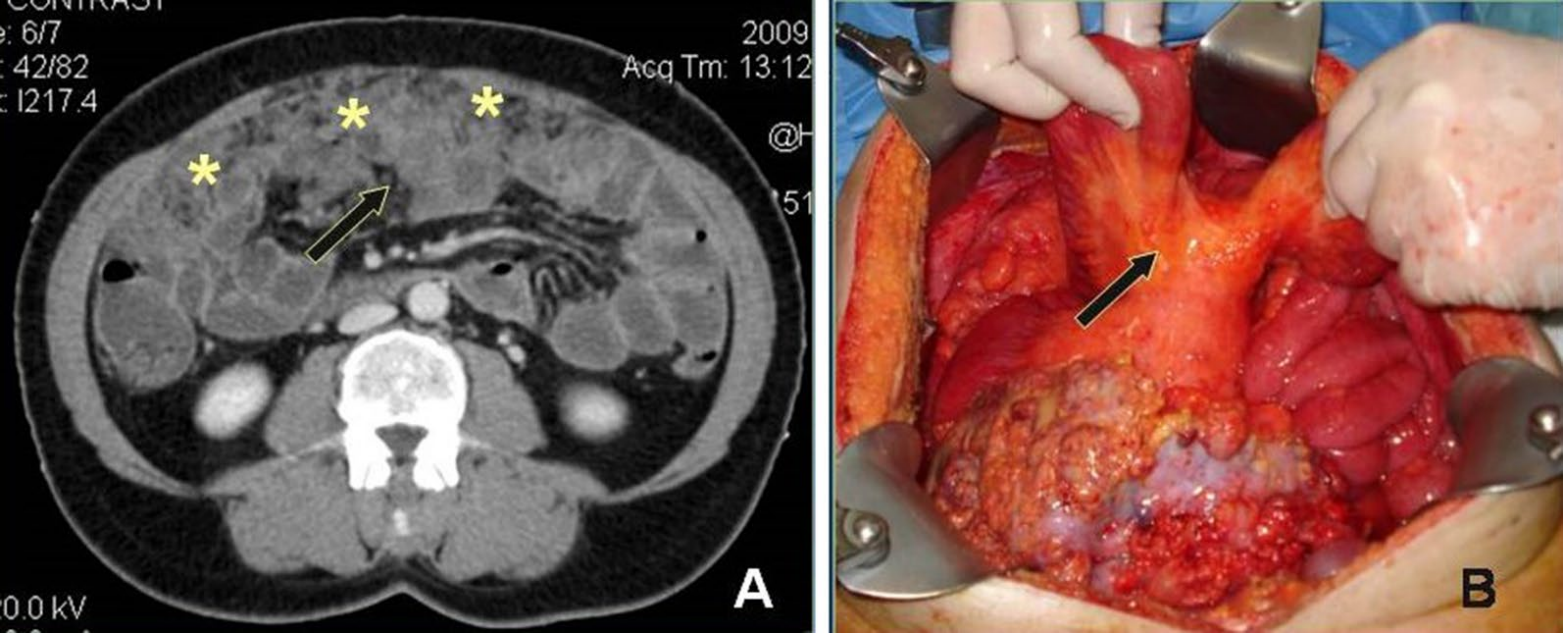
**a** Small nodules on the surface of a mesenteric fold (arrow). The omentum presents extensive infiltration (asterisks). **b** Findings at surgery verifying the presence of tiny implants (arrow) and the omental infiltration

**Fig. 13:**
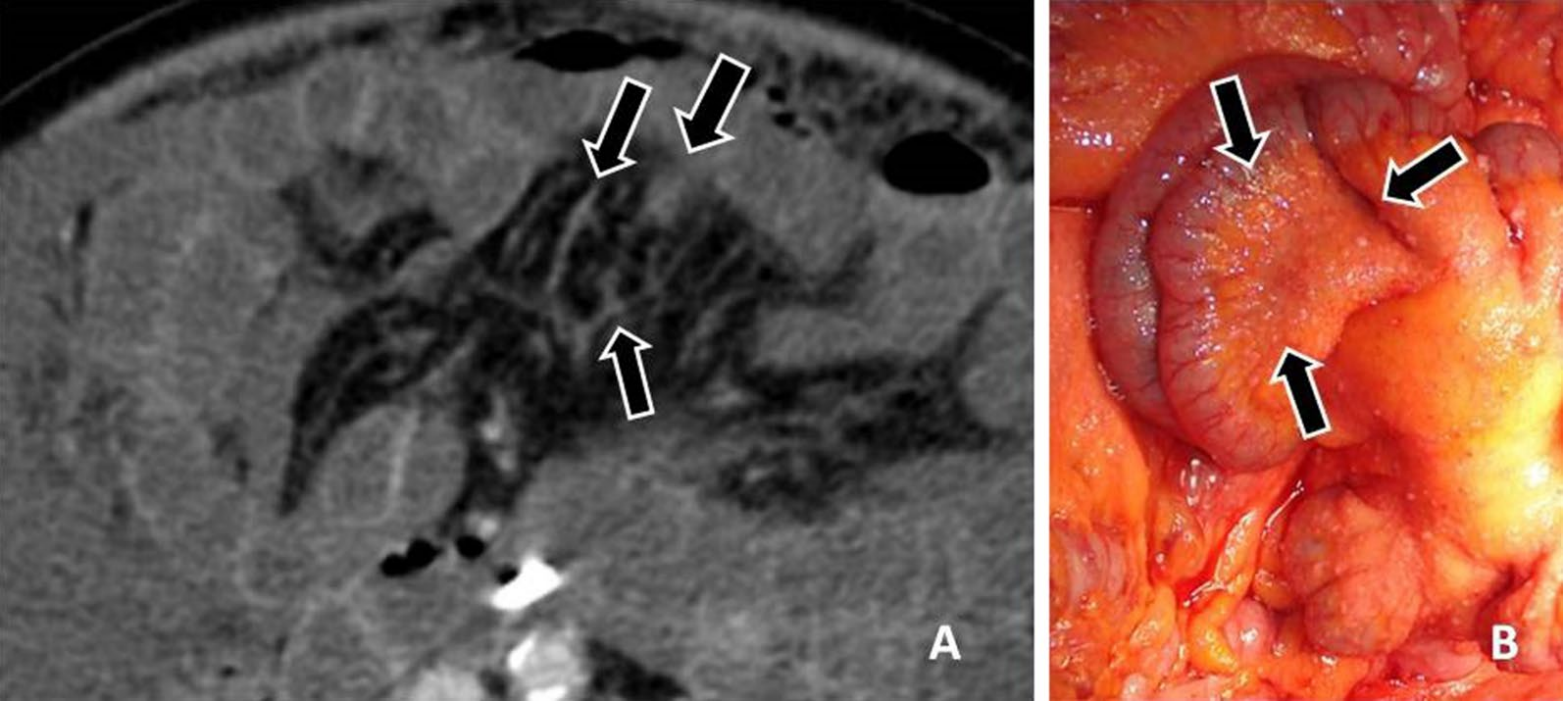
**a** CT enteroclysis image shows thickening of the mesenteric surface (arrows) and distortion and thickening of the fatty mesenteric folds. **b** At surgery multiple small implants on the mesentery are revealed (arrows)

**Fig. 14:**
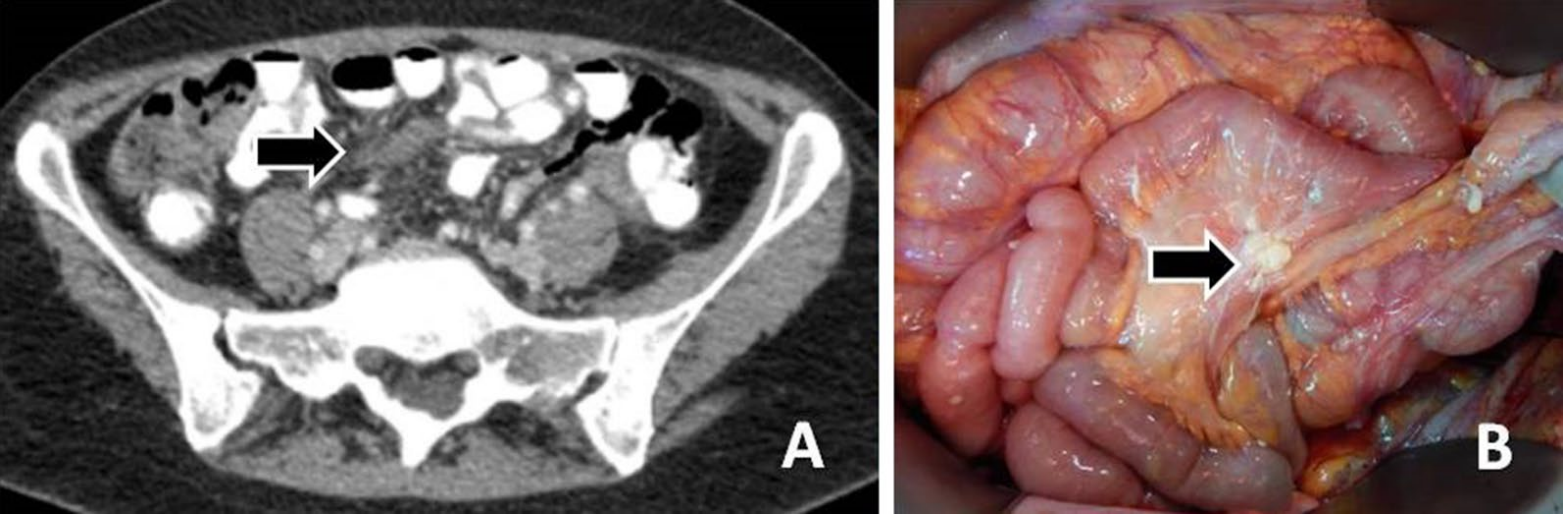
**a** Contrast-enhanced CT image shows soft tissue density lesion in the mesentery (arrow). **b** The peritoneal implant confirmed at surgery (arrow)

**Fig. 15:**
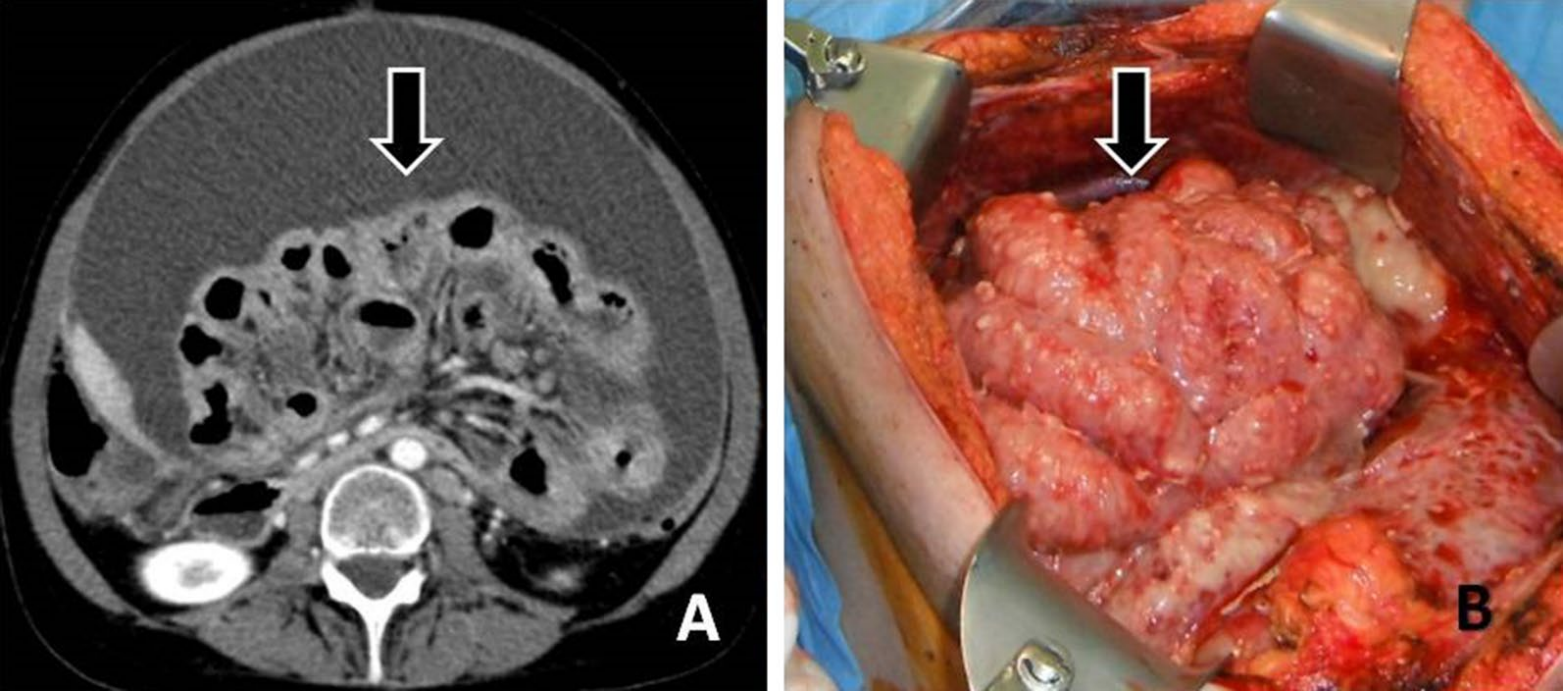
**a** Contrast enhanced CT image demonstrates shrinkage and distortion of the mesentery—“frozen mesentery”—(arrow) along with irregular thickening of the wall of intestinal loops (“layered-type” involvement of small bowel). **b** The extensively involved mesentery and intestinal loops are seen as a "cauliflower" mass at surgery (arrow)

Involvement of the SB mesentery may be seen either as increased attenuation or stranding of the mesenteric fat (Fig. 11) or nodules (Fig. 12), or soft tissue thickening of the mesenteric surface (Fig. 13) or masses (Fig. 14). Extensive involvement of SB mesentery is associated with distortion, thickening and fixation of the mesentery which has been referred to as “frozen mesentery” (Fig. 15) [5, 10, 35], considered a lethal prognostic factor.

### Malignant Pseudomyxoma peritonei

Pseudomyxoma peritonei (or “jelly belly”) is a unique form of peritoneal malignancy characterized by intraperitoneal accumulation of thick gelatinous material, due to rupture of an appendiceal or ovarian low-grade mucinous carcinoma.

**Fig. 16:**
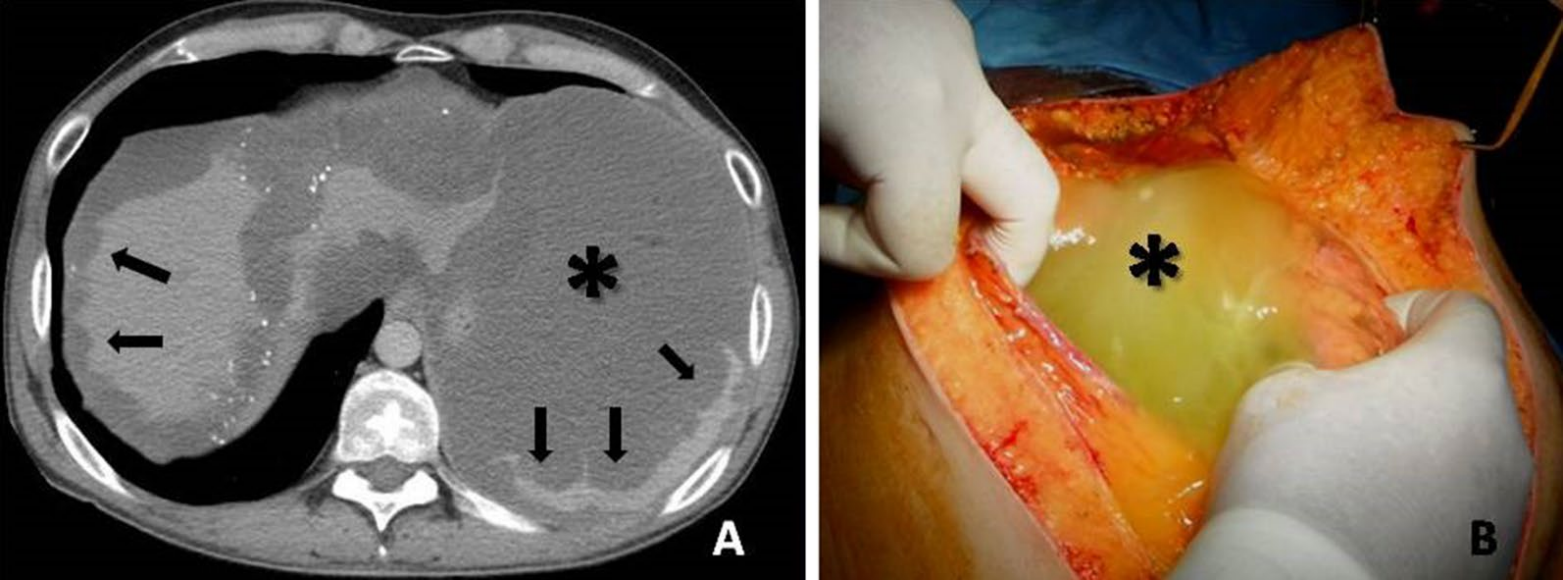
**a** Contrast-enhanced CT image reveals low-attenuation intraperitoneal gelatinous fluid (asterisk) with mass-like effect and scalloping of the surface of the liver and spleen (arrows). Multiple small calcifications are also present. **b** Intraoperative photograph shows thick gelatinous fluid filling the peritoneal cavity (asterisk)

CT shows a large amount of mass-like mucinous ascites, which produces scalloping of the visceral surfaces of the intraperitoneal organs, a finding that is most commonly observed along the margins of the liver and spleen (Fig. 16). Other CT findings include visible septa, areas of high attenuation and amorphous or curvilinear calcifications [17, 37, 38].

## Conclusion

CT has a pivotal role in the management of patients with peritoneal carcinomatosis. Thorough knowledge of the correlation between CT imaging and surgical findings is fundamental for the radiologist to reliably provide the necessary information for the appropriate selection of candidates for cytoreductive surgery and for surgical planning. Distribution of PC, “site-by-site” description of peritoneal implants, estimation of disease burden and reference to crucial sites of involvement should be included in the radiologist’s report.

## Data Availability

All data and materials are available upon request.

## Abbreviations

CRS: Cytoreductive surgery
CTE: CT Enteroclysis
HIPEC: Hyperthermic intraperitoneal chemotherapy
MDCT: Multidetector Computed tomography
MPR: Multiplanar reconstruction
PC: Peritoneal carcinomatosis
PCI: Peritoneal Cancer Index
SB: Small bowel

## References

[1] Coakley FV, Choi PH, Gougoutas CA (2002). Peritoneal metastases: detection with spiral CT in patients with ovarian cancer. Radiology.

[2] Chang MC, Chen JH, Liang JA, Huang WS, Cheng KY, Kao CH (2013) PET or PET/CT for detection of peritoneal carcinomatosis. Clin Nucl Med 38:623–629 10.1097/RLU.0b013e318299609f 23797225

[3] Kusamura S, Baratti D, Zaffaroni N (2010). Pathophysiology and biology of peritoneal carcinomatosis. World J Gastrointest Oncol.

[4] Roviello F, Caruso S, Marrelli D (2011). Treatment of peritoneal carcinomatosis with cytoreductive surgery and hyperthermic intraperitoneal chemotherapy:state of the art and future developments. Surg Oncol.

[5] Courcoutsakis N, Tentes AA, Astrinakis E, Zezos P, Prassopoulos P (2013) CT-Enteroclysis in the preoperative assessment of the small-bowel involvement in patients with peritoneal carcinomatosis, candidates for cytoreductive surgery and hyperthermic intraperitoneal chemotherapy. Abdom Imaging 38(1):56–63 10.1007/s00261-012-9869-3 22410875

[6] Klumpp BD, Aschoff P, Schwenzer N (2012). Peritoneal carcinomatosis: comparison of dymanic contrast-enhanced magnetic resonance imaging with surgical and histopathologic findings. Abdom Imaging.

[7] Aherne EA, Fenlon HM, Shields CJ, Mulsow JJ, Cronin CG (2017). What the radiologist should know about treatment of peritoneal malignancy. AJR Am J Roentgenol.

[8] Al-Shammaa HAH, Li Y, Yonemura Y (2008). Current status and future strategies of cytoreductive surgery plus intraperitoneal hyperthermic chemotherapy for peritoneal carcinomatosis. World J Gastroenterol.

[9] Dromain C, Leboulleux S, Auperin A (2008). Staging of peritoneal carcinomatosis: enhanced CT vs. PET/CT. Abdom Imaging.

[10] Nougaret S, Addley HC, Colombo PE (2012). Ovarian carcinomatosis: how the radiologist can help plan the surgical approach. Radiographics.

[11] Krishnamurthy S, Balasubramaniam R (2016). Role of imaging in peritoneal surface malignancies. Indian J Surg Oncol.

[12] Jacquet P, Jelinek JS, Steves MA (1993). Evaluation of computed tomography in patients with peritoneal carcinomatosis. Cancer.

[13] Franiel T, Diederichs G, Engelken F, Elgeti T, Rost J, Rogalla P (2009) Multi-detector CT in peritoneal carcinomatosis: diagnostic role of thin slices and multiplanar reconstructions. Abdom Imaging 34:49–54 10.1007/s00261-008-9372-z 18264738

[14] Kulinna C, Eibel R, Matzek W (2004). Staging of rectal cancer: diagnostic potential of multiplanar reconstructions with MDCT. AJR Am J Roentgenol.

[15] Pannu HK, Bristow RE, Montz FJ, Fishman EK (2003). Multidetector CT of peritoneal carcinomatosis from ovarian cancer. Radiographics.

[16] Rodolfino E, Devicienti E, Micco M (2016). Diagnostic accuracy of MDCT in the evaluation of patients with peritoneal carcinomatosis from ovarian cancer: is delayed enhanced phase really effective?. Eur Rev Med Pharmacol Sci.

[17] Coakley FV, Hricak H (1999). Imaging of peritoneal and mesenteric disease: key concepts for the clinical radiologist. Clin Radiol.

[18] Kyriazi S, Collins DJ, Morgan VA, Giles SL, deSouza NM (2010) Diffusion-weighted imaging of peritoneal disease for noninvasive staging of advanced ovarian cancer. Radiographics 30:1269–2128 10.1148/rg.305105073 20833850

[19] Meyers MA (2000) Intraperitoneal seeding: pathways of spread and localization. In: Meyers MA, Charnsangavej C, Oliphant M (ed) Meyers' dynamic radiology of the abdomen, 6th edn. Springer, New York. [Reviewer 1, comment 1]

[20] Abdalla Ahmed S, Abou-Taleb H, Ali N, Badary DM (2019). Accuracy of radiologic-laparoscopic peritoneal carcinomatosis categorization in the prediction of surgical outcome. Br J Radiol.

[21] Jacquet P, Sugarbaker P (1996). Clinical research methodologies in diagnosis and staging of patients with peritoneal carcinomatosis. Cancer Treat Res.

[22] Llueca A, Serra A, Rivadulla I, Gomez L, Escrig J (2018) Prediction of suboptimal cytoreductive surgery in patients with advanced ovarian cancer based on preoperative and intraoperative determination of the peritoneal carcinomatosis index. World J Surg Oncol 16:37. 10.1186/s12957-018-1339-0 10.1186/s12957-018-1339-0 PMC5824576 29471831

[23] Prassopoulos P, Courcoutsakis N, Tentes A (2018) Imaging of peritoneal cavity carcinoma. In: Gouliamos A, Andreou J, Kosmidis P (ed) Imaging in clinical oncology, 2nd edn. Springer, New York

[24] Flicek K, Ashfaq A, Johnson CD, Menias C, Bagaria S, Wasif N (2016) Correlation of radiologic with surgical peritoneal cancer index scores in patients with pseudomyxoma peritonei and peritoneal carcinomatosis: how well can we predict resectability? J Gastrointest Surg 20:307–312 10.1007/s11605-015-2880-6 26162922

[25] Laghi A, Bellini D, Rengo M (2017). Diagnostic performance of computed tomography and magnetic resonance imaging for detecting peritoneal metastases: systematic review and meta-analysis. Radiol Med.

[26] Sugarbaker PH, Sardi A, Brown G, Dromain C, Rousset P, Jelinek JS (2017) Concerning CT features used to select patients for treatment of peritoneal metastases, a pictorial essay. Int J Hyperth 33(5):497–504 10.1080/02656736.2017.1317368 28540832

[27] Bhatt A, Rousset P, Benzerdjeb N (2020). Prospective correlation of the radiological, surgical and pathological findings in patients undergoing cytoreductive surgery for colorectal peritoneal metastases: implications for the preoperative estimation of the peritoneal cancer index. Colorectal Dis.

[28] Schmidt S, Meuli RA, Achtari C, Prior JO (2015). Peritoneal carcinomatosis in primary ovarian cancer staging: comparison between MDCT, MRI, and 18F-FDG PET/CT. Clin Nucl Med.

[29] Ahmed SA, Abou-Taleb H, Ali N, Badary DM (2019). Accuracy of radiologic - laparoscopic peritoneal carcinomatosis categorization in the prediction of surgical outcome. Br J Radiol.

[30] Sugarbaker P (2019). Peritoneal tunnels: a site at risk for treatment failure when performing treatment for peritoneal metastases. A case series of 2 patients. Int J Surg Case Rep.

[31] Rossi A, Rossi G (2001) Diffusion of malignant tumors of intraperitoneal tumors to the peritoneum, ligaments, mesenteries, omentum and lymph nodes. In: Baert A, Sartor K (ed) CT of the peritoneum. Springer, New York

[32] Diop AD, Fontarensky M, Montoriol PF, Da Ines D (2014). CT imaging in peritoneal carcinomatosis and its mimics. Diagn Interv Imaging.

[33] Sureka B, Meena V, Garg P, Yadav T, Khera PS (2018) Computed tomography imaging of ovarian peritoneal carcinomatosis: a pictorial review. Pol J Radiol 83:500–509 10.5114/pjr.2018.80247 PMC6334186 30655930

[34] Ferrantina G, Sallustio G, Fagotti A (2009). Role of CT scan-based and clinical evaluation in the preoperative prediction of optimal cytoreduction in advanced ovarian cancer. Br J Cancer.

[35] Woodward PJ, Hosseinzadeh K, Saenger JS (2004). Radiologic staging of ovarian carcinoma with pathologic correlation. Radiographics.

[36] Levy AD, Shaw JC, Sobin LH (2009). Secondary tumors and tumorlike lesions of the peritoneal cavity: imaging features with pathologic correlation. Radiographics.

[37] Jeong YJ, Kim S, Kwak SK (2008). Neoplastic and non-neoplastic conditions of serosal membrane origin: CT findings. Radiographics.

[38] Cho JH, Kim SS (2020) Peritoneal carcinomatosis and its mimics: review of CT findings for differential diagnosis. J Belgian Soc Radiol. 104(1):8,1–6 10.5334/jbsr.1940 PMC6993595 32025624

